# Bioassay-guided isolation of antioxidant, antibacterial, and antidiabetic compounds from *Aleuritopteris bicolor* of Nepal: *In vitro/in silico* study

**DOI:** 10.1371/journal.pone.0354665

**Published:** 2026-07-30

**Authors:** Rekha Bhandari, Sadikshya Sapkota, Peru Kumari Bishwakarma, Shailendra Kumar Sharma, Ram Kishor Yadav, Sandesh Poudel, Siddha Raj Upadhyaya, Ganga Ram Upadhayay, Sajan L. Shyaula, Khem Raj Joshi

**Affiliations:** 1 School of Health and Allied Sciences, Pokhara University, Pokhara, Nepal; 2 Central Department of Chemistry, Tribhuvan University, Katmandu, Nepal; 3 Faculty of Science, Nepal Academy of Science and Technology, Lalitpur, Nepal; Banaras Hindu University, INDIA

## Abstract

*Aleuritopteris bicolor*, a fern species known locally as “Raani Sinkaa” in Nepal, has traditionally been used to treat wounds, diarrhea, dysentery, and gastritis. Despite its widespread use, scientific data on its bioactive compounds and bioactivities remain limited. To address this gap, this study aimed to isolate bioactive compounds from *A. bicolor* and evaluate their antioxidant, antibacterial, and antidiabetic properties using both *in vitro* and *in silico* methods. The 70% methanol extract of *A. bicolor* afforded three compounds: 20-hydroxyecdysone (**1**), and a mixture of quercetin 3-*O*-*β*-D-glucopyranoside (**2**) and quercetin 3-*O*-*β*-D-galactopyranoside (**3**), isolated via column chromatography. The structures of the isolated compounds were elucidated based on spectroscopic (NMR) and spectrometric (LC-MS) analyses. Compound **1** exhibited weak antioxidant activity, with an IC_50_ of 38.38 ± 3.41 µg/mL in the DPPH free radical scavenging assay. In contrast, the mixture of compounds **2** and **3** demonstrated significant antioxidant activity, with an IC_50_ of 1.56 ± 0.3 µg/mL, surpassing the standard ascorbic acid (IC_50_: 4.15 ± 0.3 µg/mL). Compounds **2** and **3** also showed moderate antibacterial activity against *Staphylococcus aureus* and *Klebsiella pneumoniae*. Furthermore, these compounds exhibited notable inhibition of *α*-amylase and *α*-glucosidase enzymes in *in vitro* assays, with EC_50_ values of 99.48 ± 3.2 µg/mL and 68.53 ± 1.7 µg/mL, respectively. *In silico* molecular docking analyses supported these enzyme inhibitions by revealing favorable interactions between compound **2** and key catalytic residues of the target enzymes, with binding energies of −7.2 kcal/mol for *α*-amylase and −7.1 kcal/mol for *α*-glucosidase. Additionally, ADMET analysis suggests further lead optimization of compounds **2** and **3**. This study successfully isolated three bioactive compounds from *A. bicolor* and recommends further medicinal chemistry and *in vivo* investigations to establish their therapeutic potential as antidiabetic phytomedicine.

## 1. Introduction

Human civilization has long harnessed the therapeutic potential of plants to treat various ailments. Today, approximately 60% of the global population, including 80% in developing nations, relies on herbal medicine as a primary healthcare resource due to its perceived safety, affordability, and accessibility [1]. Phytochemicals derived from medicinal plants offer a diverse array of bioactive compounds capable of modulating multiple biological targets, making them valuable leads in drug discovery and development [2–4]. As the herbal medicine market continues to expand—exceeding a trillion dollars with an annual growth rate of over 8%—the exploration of botanicals with traditionally proven pharmacological benefits remains a critical area of research [5].

Oxidative stress resulting from excess reactive oxygen species (ROS) contributes to many chronic diseases, including diabetes, cancer, and cardiovascular conditions [6–9]. Plant-derived antioxidants such as phenolics and flavonoids have the potential to mitigate this damage and promote health [6,10]. Additionally, the rise of antimicrobial resistance (AMR) threatens global health, making the search for new natural antimicrobial agents from medicinal plants essential, as many contain bioactive compounds with broad-spectrum antibacterial activity [11–13]. Furthermore, diabetes mellitus affects over 536 million people worldwide, with numbers projected to increase further [14]. Current antidiabetic drugs often have limitations, including side effects, prompting the need for safer, plant-based alternatives. Inhibition of carbohydrate-hydrolyzing enzymes such as *α*-amylase and *α*-glucosidase is a proven strategy to control postprandial hyperglycemia, and many medicinal plants have shown promise in this regard [11,15,16]. Therefore, investigating the antioxidant, antimicrobial, and antidiabetic properties of medicinal plants offers a valuable pathway for discovering novel therapeutic compounds.

*Aleuritopteris bicolor* (Roxb.) Fraser-Jenk (synonym: *Cheilanthes bicolor*), an edible fern of the Pteridaceae family, grows in moist habitats such as rocky areas exposed to sunlight and shaded forests. This species is native to Nepal, India, China, Bangladesh, Sri Lanka, and Pakistan. In Nepal, it is referred to as Kali Sinki or Raani Sinka and is traditionally used to treat sinusitis, fever, cuts, as well as to manage diarrhea, dysentery, diabetes, and gastritis [17,18]. Previous studies have identified various classes of phytochemicals in *A. bicolor* extracts, including phenolics, flavonoids, terpenoids, and glycosides [17,18]. Likewise, Jha et al. have demonstrated notable antioxidant, anti-inflammatory, and *α*-amylase inhibitory activities of ethanolic extracts of *A. bicolor* [17,19].

Despite its ethnopharmacological significance and preliminary reports of diverse phytochemicals and biological activities, the scientific exploration of its bioactive compounds and therapeutic efficacies remains limited as of January 2025. To address this gap, this study aimed to isolate and characterize bioactive compounds from *A. bicolor* and evaluate their antioxidant, antibacterial, and antidiabetic activities. Employing bioassay-guided fractionation, along with *in vitro* assays, *in silico* molecular docking, and ADMET profiling, this research seeks to substantiate the traditional uses of *A. bicolor* and explore its potential as a natural antidiabetic remedy, thereby promoting evidence-based phytomedicine practices.

## 2. Methods

### 2.1. Chemicals, enzymes and bacterial strain

MCI gel CHP20P (75 ~ 150 *μ*m) (Mitsubishi Chemical Industries Co., Ltd., Japan); Sephadex LH-20 (Amersham Pharmacia Biotech, Sweden); Chromatorex ODS (30 ~ 50 μm) (Fuji Silysia Chemical Co., Ltd., Japan); TLC silica gel 60 F_254_ (Sigma-Aldrich, Germany); methanol, ascorbic acid (Merck, India); Mueller Hinton agar, amikacin discs, meropenem discs, quercetin dihydrate (HiMedia, India); 2,2-diphenyl-1-picrylhydrazyl (DPPH) and enzymes *α*-amylase and *α*-glucosidase (Sigma-Aldrich, USA) were used in the current investigation. Bacterial strains, including *Staphylococcus aureus*, *Klebsiella pneumoniae*, and *Escherichia coli*, were obtained from Manipal Teaching Hospital, Pokhara, Nepal.

### 2.2. Plant material

On May 25th, 2024, *A. bicolor* was collected from the hills of Rupa Lake (Latitude: 28.1542° N, Longitude: 84.1132° E, Altitude: 950 m), Pokhara Metropolitan City, Kaski District, Nepal. The collection was carried out with the landowner’s consent and in full compliance with national guidelines, including the Convention on International Trade in Endangered Species of Wild Fauna and Flora (CITES) and the policies of the International Union for Conservation of Nature (IUCN). Since *A. bicolor* is not an endangered or protected species in Nepal, and the sampling was solely for academic research purposes, no special government permit was required. Its identity was confirmed by botanist Dhan Raj Kandel, a research officer at the National Herbarium and Plant Laboratories, Godawari-3, Lalitpur, Nepal.

### 2.3. Bioassay-guided isolation

The extraction and isolation of the chemical compound from the 70% MeOH extract of *A. bicolor* were carried out in accordance with previous methods [20,21]. The shade-dried aerial parts of *A. bicolor* (400 g) were first extracted with 5 L of 70% MeOH (2 hours at 55 °C and 22 hours at room temperature), followed by extraction with 4 L of 70% MeOH (24 hours at room temperature). The extracts were filtered, combined, and evaporated under reduced pressure at 55°C using a rotary evaporator (Biobase RE-2000B, Germany) to obtain 111.8 g of dry extracts. The dry extract (111.8 g) was stirred with distilled water and allowed to stand undisturbed for 10 minutes. The mixture was then filtered to obtain water-soluble fraction (filtrate: 60.6 g) and the water-insoluble fraction (residue: 51.2 g). The water-soluble fraction (60.6 g) was subjected to column chromatography on MCI gel CHP20P (bed volume 600 cm^3^) and eluted with H_2_O, 40% MeOH, 70% MeOH, MeOH, and chloroform, yielding nine fractions (1 ~ 9). Based on the separation pattern of DPPH-active antioxidant compounds observed in TLC, fractions 6 was further subjected to additional column chromatography.

Fraction 6 (1150 mg, 40% MeOH eluate) was subjected to Sephadex LH-20 column chromatography (bed volume 400 cm^3^) and eluted with MeOH to obtain two subfractions (6−1 and 6−2). Subsequently, subfraction 6−1 (660 mg) was subjected to silica column chromatography (bed volume 500 cm^3^) and eluted with CHCl_3_: MeOH: H_2_O = 8: 2.5: 0.2) to afford compound **1** (47.7 mg). Similarly, subfraction 6−2 (240 mg) was subjected to silica gel column chromatography (CHCl_3_: MeOH: H_2_O; = 8: 3: 0.5) to afford a mixture of compound**s 2** and **3** (22.5 mg)

^1^H and ^13^C NMR spectra of the isolated compounds were recorded in DMSO-*d*_6_ using a Bruker ADVANCE 400 MHz NMR spectrometer (^1^H-NMR: 400 MHz; ^13^C-NMR: 100 MHz). The chemical shift values (*δ*_H_ and *δ*_C_) are expressed in ppm. Additionally, the molecular weight of compound was determined by liquid chromatography-mass spectrometry (LC-MS) analysis on a Shimadzu LC-MS 2020 system [8].

### 2.4. Antioxidant activity

The antioxidant activity of the isolated compounds was evaluated using the reliable DPPH free radical scavenging assay. This assay assesses the ability of the compounds to neutralize DPPH radicals via hydrogen atom donation, resulting in a color change from violet to yellow that is quantitatively measured by absorbance at 517 nm [11,22]. In brief, 1.5 mL of the test compound at varying concentrations was mixed with 1.5 mL of a 100 *µ*M DPPH methanolic solution in a microtiter plate. The mixture was incubated in darkness for 30 minutes, after which the absorbance of the oxidized DPPH radical (DPPH^**•**^) was measured at 517 nm. Ascorbic acid, ranging from 0.6125 µg/mL to 10 µg/mL, served as the standard antioxidant. The percentage of free radical scavenging activity was calculated by averaging the absorbance values from triplicate measurements against the DPPH control solution, which contained 1.5 mL of distilled water instead of the test sample. A linear regression of the percentage DPPH scavenging activity against the compound concentration was used to determine the IC_50_ value, indicating the concentration required to scavenge 50% of the free radicals. Lower IC_50_ values reflect higher antioxidant potency [11,23].


$$\begin{array}{c} \%\text{ DPPH radical scavenging}=\left[\left({\text{A}}_{\text{0}}-{\text{A}}_{\text{1}}\right)/{\text{A}}_{\text{0}}\right]\times \text{100}. \end{array}$$

where A_0_ is the absorbance of the DPPH^•^ control, and A_1_ is the absorbance of the test sample or reference sample.

### 2.5. Antibacterial activity

The antibacterial efficacy of the isolated compounds was assessed using the well diffusion method against three bacterial strains obtained from the American Type Culture Collection: *Staphylococcus aureus* (ATCC 11238), *Klebsiella pneumoniae* (ATCC 70065), and *Escherichia coli* (ATCC 11386), following the protocol established in our prior study [11,23]. Standardized bacterial suspensions, equivalent to 0.5 McFarland turbidity, were uniformly swabbed onto the surface of Muller-Hinton agar plates. Subsequently, five wells, each with a diameter of 6 mm, were aseptically bored into each agar plate. To each well, 100 μL of the isolated compounds at concentrations of 12.5, 25, and 50 µg/mL were added. The plates were then incubated at 37°C for 48 hours. A 5% dimethyl sulfoxide (DMSO) solution served as the negative control, while an amikacin disc (30 μg) was used as the positive control. After incubation, the zones of inhibition (ZOI) surrounding the wells were measured in millimeters to quantify antibacterial activity.

### 2.6. Antidiabetic activity

#### 2.6.1. *α*-amylase inhibition assay.

The *α*-amylase inhibitory potential of isolated compounds was estimated following a previously established protocol [11,24]. In this assay, *α*-amylase enzymatically hydrolyzed the substrate 2-chloro-4-nitrophenyl-*α*-D-maltotrioside (CNPG3) into 2-chloro-4-nitrophenol (CNP), 2-chloro-4-nitrophenyl-*α*-D-maltoside (CNPG2), maltotriose, and glucose. The resulting yellow color CNP was quantified spectrophotometrically at 405 nm. Notably, substantial enzyme inhibition reduced CNP formation, leading to decreased absorbance measured by UV-VIS spectrophotometry.

Briefly, 20 µL of isolated compounds at concentrations ranging from 15 to 250 µg/mL were incubated with 80 µL of porcine pancreatic *α*-amylase enzyme solution (1.5 U/mL prepared in 50 mM phosphate-buffered saline, pH 7.0) in a 96-well microtiter plate. After a 15-minute incubation at 37 °C, the enzymatic reaction was initiated by introducing CNPG3 at a concentration of 375 µM, followed by an additional 15 minutes of incubation at 37 °C. Subsequently, the absorbance of the reaction product was measured at 405 nm using a microplate spectrophotometer. The percentage of enzyme inhibition was determined using the following formula:


$$\begin{array}{c} \alpha -\text{Amylase inhibition }(\%)=\left({\text{A}}_{\text{control}}-{\text{A}}_{\text{sample}}/{\text{A}}_{\text{control}}\right)\times \text{100} \end{array}$$

where A represent the absorbance of the sample and control.

#### 2.6.2. *α*-glucosidase inhibition assay.

The assessment of *α*-glucosidase inhibitory activity was conducted following the established protocol [11,25]. Briefly, 20 µL of isolated compounds at varying concentrations (10–1000 µg/mL) were incubated with 80 µL of *α*-glucosidase enzyme (1.5 U/mL in 50 mM phosphate-buffered saline, pH 7.0) within a 96-well microplate. After a 15-minute incubation at 37 °C, the substrate *p*-nitrophenyl-*α*-D-glucopyranoside (*p*NPG) at 375 µM was introduced to initiate the enzymatic reaction, which proceeded for an additional 15 minutes at 37 °C. Subsequently, the absorbance of the reaction product was recorded at 405 nm using a microplate reader. The inhibitory effect was quantitatively determined using the following formula:


$$\begin{array}{c} \alpha -\text{Glucosidase inhibition }(\%)=\left({\text{A}}_{\text{control}}-{\text{A}}_{\text{sample}}/{\text{A}}_{\text{control}}\right)\times \text{100} \end{array}$$

where A represent the absorbance of the sample and control.

### 2.7. *In Silico* study

#### 2.7.1. Molecular docking.

**2.7.1.1 Ligand and receptor design:** In this molecular docking investigation, *α*-amylase and *α*-glucosidase enzymes were selected as molecular targets to assess antidiabetic potential of *A. bicolor*, following validated methodologies [11]. The three-dimensional crystal structures of these proteins (PDB IDs: 4W93 and 5KZW) were obtained from the RCSB Protein Data Bank (https://www.rcsb.org/) [26–28]. Natural *α*-amylase and *α*-glucosidase inhibitors isolated from *A. bicolor* were used as ligands. Their 3D conformations, along with the reference antidiabetic compound acarbose, were downloaded in SDF format from the PubChem repository and subsequently converted to PDB format using BIOVIA Discovery Studio Visualizer. Target receptor and ligand structures were prepared by removing extraneous molecules, adding polar hydrogens, and assigning Kollman charges. These structures were then transformed into pdbqt format using AutoDock 1.5.6 software to facilitate docking studies.

**2.7.1.2 Validation of target receptors:** The structural fidelity and quality of the target enzymes were validated by Ramachandran plot analysis using the PROCHECK server (https://saves.mbi.ucla.edu/), in accordance with established protocols to ensure stereochemical reliability [11,29].

**2.7.1.3. Molecular docking:** Docking study was performed using AutoDock Vina version 1.5.7 [23]. For *α*-amylase, a cubic grid box with dimensions 20 × 20 × 20 Å was centered at coordinates x = −9.6, y = 4.4, z = −22.9, with a grid spacing of 0.375 Å. Likewise, *α*-glucosidase docking employed an identically sized grid box centered at x = −13.7, y = −19.6392, and z = −31.94, also with a spacing of 0.375 Å. These grid parameters encompassed the entire active site of each enzyme, permitting comprehensive ligand binding exploration [11]. Post-docking analyses of ligand–protein interactions were performed through BIOVIA Discovery Studio Visualizer 2020 to elucidate binding modes.

**2.7.1.4. Docking protocol validation:** The accuracy of the docking procedure was validated by calculating the root mean square deviation (RMSD) between the docked ligands and their respective native co-crystallized conformations. The native ligands were re-docked into their protein binding sites, and the resulting poses were superimposed with the original structures using PyMOL 2.5.2. An RMSD below 2 Å was indicative of a robust docking protocol, whereas values exceeding 4 Å suggested diminished reliability [29].

#### 2.7.2. ADME-toxicological assessment.

Pharmacokinetic and toxicity profiles of the isolated constituents were predicted using computational tools. SwissADME (http://www.swissadme.ch/index.php) was employed to evaluate biopharmaceutical parameters *in vivo*, including physicochemical properties, lipophilicity, aqueous solubility, pharmacokinetics, and drug-likeness metrics [11]. Additionally, toxicity risks—such as AMES mutagenicity, hepatotoxicity, nephrotoxicity, carcinogenic potential, cytotoxicity, and mutagenicity—were predicted using ProTox-3.0 (https://comptox.charite.de/protox3/) to assess safety liabilities [21].

### 2.8. Statistical analysis

Statistical analyses were conducted using Microsoft Excel 2016. Each experiment was performed in triplicate, and results are expressed as mean ± standard deviation. Antioxidant activity (IC_50_) and antidiabetic enzyme inhibitory activity (EC_50_) were evaluated using linear regression analysis. Student’s t-test was employed to determine statistical significance at p < 0.05.

## 3. Results and discussion

### 3.1. Spectroscopic analysis and structure elucidation

**Fig 1:**
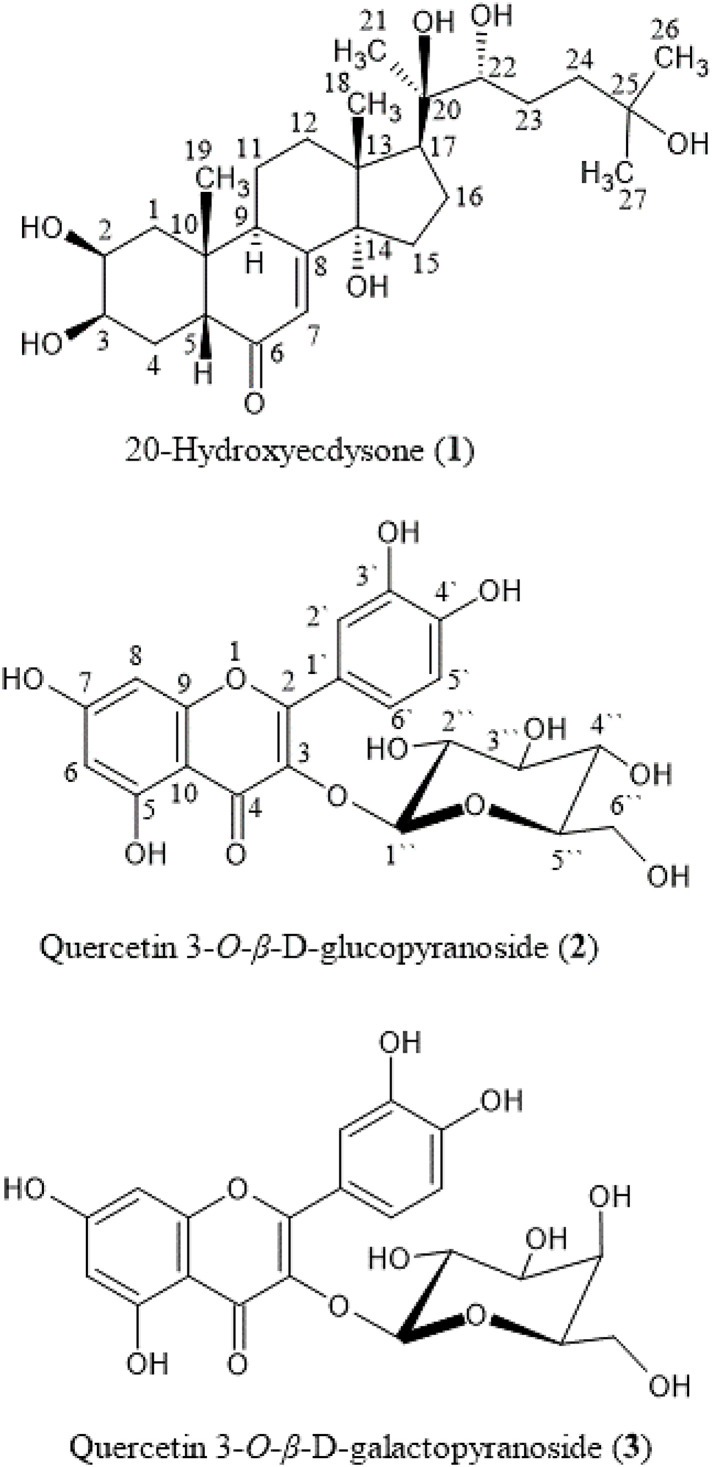
Bioactive compounds isolated from 70% MeOH extract of *A. bicolor.*

Compound **1** (C_27_H_44_O_7;_ molecular weight – 480.6 g/mol) was obtained as white powder soluble in methanol and DMSO. TLC: Rf = 0.9 (CHCl_3_: MeOH: H_2_O; = 8:2:0.2), visible at 254 nm and invisible at 365 nm under UV light, negative towards FeCl_3,_ positive towards H_2_SO_4_/heat and gives a black color band, and pale-yellow band towards DPPH suggesting antioxidant properties. The ^1^H NMR spectrum of compound **1** in DMSO-*d*_6_ (S1 Fig in S1 File) is detailed in Table 1. The ^13^C-NMR spectra of compound **1** in DMSO-*d*_6_ (S2 Fig in S1 File) showed signals equivalent to a total of 27 carbons (Table 1). The DEPT-135 NMR spectra (S3 Fig in S1 File) showed positive signals at *δ*_C_ 120.9 (C-7), 76.7 (C-22), 67.2 (C-2), 67.1 (C-3), 50.5 (C-5), 49.2 (C-17), 33.6 (C-9), 29.8 (C-26), 29.4 (C-27), 24.3 (C-19), 21.4 (C-21) and 17.3 (C-18) with the absence of a peak at *δ*_C_ 202.8 (C-6), 165.3 (C-8), 83.1 (C-14), 75.8 (C-20), 68.8 (C-25), 46.9 (C-13), 41.4 (C-24) and 37.7 (C-10), confirming the presence of a carbonyl carbon (C-6) and seven quaternary carbon. Similarly, the negative signals at *δ*_C_ 41.8 (C-24), 37.0 (C-1), 31.9 (C-4), 30.8 (C-15), 26.7 (C-11), 26.5 (C-23) 20.8 (C-16) indicated the presence of the seven CH_2_ group. Furthermore, ESI/MS of compound **1** revealed mass spectra at *m/z* 479.70 [M-H]^-^, 515.68 [M + Cl]^-^, and 525. 72 [M+HCOO]^-^ in negative mode ESI. By comparing these data with those reported in literature of fern species [30], compound **1** was elucidated as 20- hydroxyecdysone for the first time from *A. bicolor,* as illustrated in Fig 1.

**Table 1:** ^1^H, ^13^C, and DEPT-NMR spectroscopic data of compound 1 and the reference 20-hydroxyecdysone in DMSO-*d*_6._

Position	Compound 1			20-hydroxyecdysone	
	*δ*_H,_ mult. (*J* in Hz)	*δ*_C_	DEPT-135	*δ*_H_, mult. (*J* in Hz)	*δ*_C_
1	1.54-1.57, m	36.7	37	1.52, m	36.6
	1.22-1.28, m			1.28, m	
2	3.57, brs	66.9	67.2	3.57, brs	66.7
3	3.76, s	66.7	67.1	3.75, brs	66.5
4	1.54-1.57, m	31.6	31.9	1.57, m	31.5
	1.54-1.57, m			1.51, m	
5	2.18, dd (13.2, 15.6)	50.2	50.5	2.19, dd (3.6,13.2)	50.1
6		202.8			202.6
7	5.62, s	120.5	120.9	5.61, brs	120.4
8		165.3			165.2
9	2.99, brs	33.2	33.6	2.99, dd (3.6,13.2)	33.1
10		37.7			37.6
11	1.61-1.7, m	20.2	20.7	1.68, m	20
	1.54-1.57, m			1.50, m	30.8
12	2, t (11.6)	30.9	30.8	2, m	30.8
	1.61-1.7, m			1.69, m	
13		46.9			46.8
14		83.1			82.9
15	1.73-1.77, m	30.9	30.8	1.79, m	30.8
	1.48, m			1.49, m	17.1
16	1.85-1.87, m	20.3	20.8	1.87, m	20.2
	1.54-1.57, m			1.53, m	
17	2.24	48.8	49.2	2.24, t (9.2)	48.6
18	0.75, s	17.2	17.3	0.75, s	17.1
19	0.82, s	23.9	24.3	0.82, s	23.8
20		75.8			75.6
21	1.06, s	21	21.4	1.04, s	20.9
22	3.10, t (8.4)	76.3	76.7	3.10, t (7.6)	76.1
23	1.48, m	26.1	26.5	1.46, m	26
				1.08, m	
24	1.61-1.7, m	41.4	41.8	1.6, m	41.4
	1.22-1.28, m			1.25, m	
25		68.8			68.6
26	1.61-1.7, m	29.4	29.8	1.6, s	30
27	1.04, s	29.1	29.4	1.03, s	28.9
					
2-OH	4.45	–	–	4.38, d (2.4)	–
3-OH	4.45	–	–	4.39, d (6)	–
14-OH	4.64, s	–	–	4.70, s	–
20-OH	3.56, s	–	–	3.58, s	–
22-OH	4.35, d (12)	–	–	4.35, d (4.8)	–
25-OH	4.12, s	–	–	4.12, s	–

Compound **2 and 3** (mixture) (C_21_H_20_O_12;_ molecular weight – 464.4 g/mol) was obtained as yellow amorphous powder soluble in methanol and DMSO. TLC: Rf = 0.4 (CHCl_3_: MeOH: H_2_O; = 8:2:0.2), visible at 254 nm and 365 nm under UV light, positive (black color) towards FeCl_3,_ positive (orange color) towards H_2_SO_4_/heat suggesting flavonoids, and yellow band towards DPPH suggesting antioxidant properties. The ^1^H (S4 Fig in S1 File), ^13^C (S5 Fig in S1 File), DEPT-135 (S6 Fig in S1 File) NMR spectrum of compound **2** and **3** in DMSO-*d*_6_ is detailed in Table 2. ESI/MS of compound **2** revealed mass spectra at *m/z* 463.44 [M-H]^-^, 499.45 [M + Cl]^-^, and 927.68 [2M-H]^-^ in negative mode ESI. By comparing these data with those reported in literature of same genus [31,32], compounds **2** and **3** were elucidated as quercetin 3-*O*-*β*-D-glucopyranoside and quercetin 3-*O*-*β*-D-galactopyranoside for the first time from *A. bicolor* (Fig 1).

**Table 2:** ^1^H, ^13^C, and DEPT-NMR data of compounds 2, 3 and the reference quercetin 3-*O*-*β*-D-glucopyranoside, and quercetin 3-*O*-*β*-D-galactopyranoside in DMSO-*d*_6._

Position	Compound 2			Quercetin 3-*O*-*β*-D-glucopyranoside		Compound 3			Quercetin 3-*O*-*β*-D-galactopyranoside	
	*δ*_H,_ mult. (*J* in Hz)	*δ*_C_	DEPT-135	*δ*_H,_ mult. (*J* in Hz)	*δ*_C_	*δ*_H,_ mult. (*J* in Hz)	*δ*_C_	DEPT-135	*δ*_H,_ mult. (*J* in Hz)	*δ*_C_
1										
2		156.4			156.3		156.3			156.2
3		133.4			133.4		133.5			133.5
4		177.5			177.5		177.5			177.5
5		161.3			161.2		161.3			161.2
6	6.19, d (2.2)	98.7	99.1	6.19, d (2.2)	98.7	6.40 d (2.4)	98.7	99.1	6.40, d (2.4)	98.7
7		164.3			164.3		164.3			164.1
8	6.4, d (2.2)	93.6	93.9	6.38, d (2.2)	93.5	6.19	93.6	93.9	6.19, d (2.4)	93.4
9		156.2			156.2		156.2			156.3
10		104			104		103.9			103.9
1`		121.2			121.2		121.1			121.1
2`	7.52, d (2.2)	115.2	115.6	7.52, d (2.2)	115.2	7.55, d (2.4)	115.2	115.6	7.53, d (2.4)	115.2
3`		144.9			144.8		144.9			144.8
4`		148.5			148.5		148.5			148.5
5`	6.81, d (8.4)	116.3	116.6	6.81, d (8.4)	116.2	6.83, d (8.4)	116.0	116.4	6.81, d (8.5)	115.9
6`	7.52, brd	121.7	121.1	7.54, brd (8.4)	121.6	7.65, dd (2.4, 8)	122.0	122.4	7.66, dd (2.4, 8.5)	122
C_5_-OH	12.62, brs			12.62, brs		12.62, brs			12.62, brs	
Gal/Glc 1	5.36, d (7.2)	100.9	101.3	5.34, d (7.3)	100.9	5.45	101.8	102.2	5.37, d (7.9)	101.8
Gal/Glc 2	3.08-3.31	74.2	74.5	3.05-3.31	74.1	3.57, d (9.6)	71.2	71.6	3.56, dd (7.9, 9.2)	71.2
Gal/Glc 3	3.08-3.31	77.6	78	3.05-3.31	77.5	3.08-3.65	73.2	73.6	3.41-3.45	73.2
Gal/Glc 4	3.08-3.31	70	70.3	3.05-3.31	69.9	3.08-3.66	68	68.3	3.41-3.45	67.9
Gal/Glc 5	3.08-3.31	76.5	76.9	3.05-3.31	76.5	3.08-3.67	75.9	76.2	3.29, ddd (6.1, 10.1, 10.7)	75.8
Gal/Glc 6	3.6	61	61.4	3.67, d (10.2) 3.22, m	61	3.08-3.68	60.2	60.5	3.45, dd (7.3, 10.7)	60.1

### 3.2. Antioxidant activity

**Fig 2:**
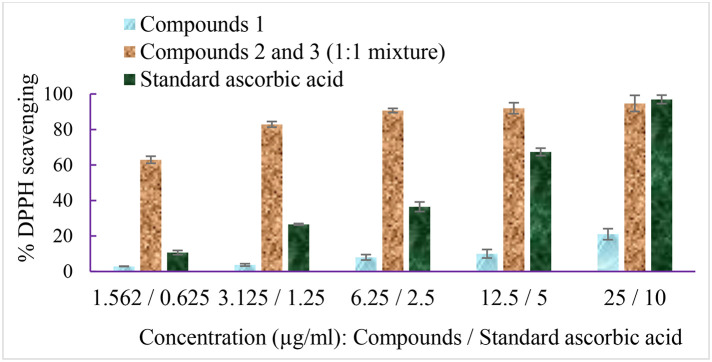
Antioxidant activity of isolated compounds and standard ascorbic acid assessed via DPPH free radical scavenging assay. Each test was performed in triplicate (n = 3) and the data was expressed in terms of IC_50_ (mean ± S.D).

**Fig 3:**
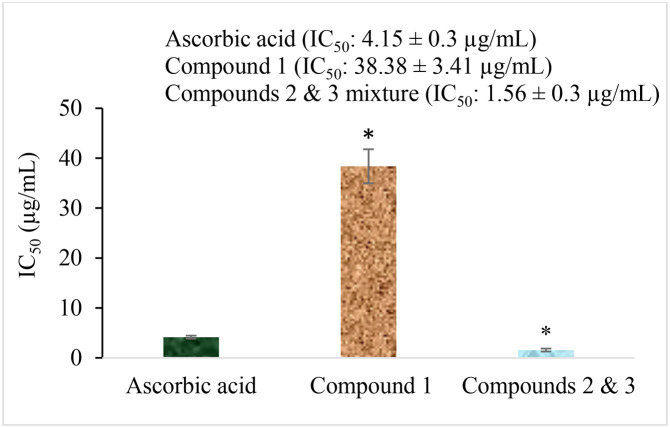
Fifty percentage DPPH free radical scavenging concentration (IC_50_) of isolated compounds and standard ascorbic acid. Lower values of IC_50_ indicates strong antioxidant activity. *Significantly different from standard antioxidant (ascorbic acid) at p < 0.05, using Student’s t-test.

Compound **1** exhibited weak antioxidant activity (IC_50_: 38.38 ± 3.41 µg/mL). Meanwhile, compounds **2** and **3** showed significant antioxidant activity (*p* < 0.05), with IC_50_ value of 1.56 ± 0.3 µg/mL, surpassing the standard ascorbic acid (IC_50_: 4.15 ± 0.3 µg/mL), as illustrated in Fig 2 and Fig 3. In prior studies, 20-hydroxyecdysone (**1**) displayed an IC_50_ of 155.82 ± 9.82 µg/mL in DPPH assay [33]. Likewise, Joshi et al. [32] reported the DPPH scavenging activity of quercetin 3-*O*-*β*-D-glucopyranoside (**2**) and quercetin 3-*O*-*β*-D-galactopyranoside (**3**), with DPPH IC_50_ values of 54.6 *µ*M, and 51.4 *µ*M, respectively. The significant antioxidant properties of compounds **2** and **3** are attributed to the presence of free phenolic hydroxyl groups (C5-OH, C7-OH, C3′-OH, and C4′-OH), as supported by the structure-activity relationship of antioxidant polyphenols [32]. These phyto-antioxidants protect human tissues against oxidative stress and damage caused by free radicals and reactive oxygen species [34], highlighting the significant potential of *A. bicolor* for inclusion in drug discovery and development pipelines targeting oxidative stress-related diseases such as diabetes, cancer, and inflammatory conditions.

### 3.3. Antibacterial activity

**Fig 4:**
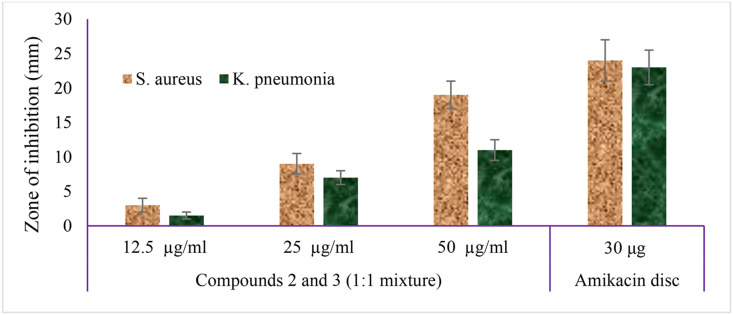
Antibacterial activity of isolated compounds and standard antibiotic meropenem through disc diffusion assay. Each test was performed in triplicate (n = 3) and the data was expressed in terms of ZOI (mean ± S.D).

Compounds **2** and **3** demonstrated antibacterial effect against Gram-positive *S. aureus* and *K. pneumonia* in the well diffusion assay, producing inhibition zones of 19 and 13 mm, respectively, at the concentration of 50 µg/mL. In comparison, the standard antibiotic meropenem exhibited zone of inhibition (ZOI) of 24 and 22 mm (Fig 4). These findings are consistent with several previous studies [35–38]. Conversely, compound **1** did not exhibit any antibacterial activity. Additionally, all isolated compounds lacked activity against Gram- negative strain *E. coli*. Ben et al., on the contrary, claimed active antibacterial activity status of 20-hydroxyecdysone (**1**) against *P. aeruginosa, E. coli*, *S. aureus*, and *E. faecalis,* estimating minimum inhibitory concentration (MIC) values of 0.062 ± 0.001 mg/mL, 0.125 ± 0.010 mg/mL, 0.125 ± 0.011 mg/mL, and 0.125 ± 0.016 mg/mL, respectively [33].

Several previous studies have demonstrated that flavonoids, particularly quercetin, exhibit antibacterial effects by disrupting bacterial cell membranes through hydrogen bonding. They also inhibit cell wall synthesis, biofilm formation, enzyme activity, and ATP production, ultimately leading to bacterial death [36–40]. The resistance observed in *E. coli* is likely due to the protective lipopolysaccharide layer in their outer membrane, which impedes the diffusion of antibacterial agents into these Gram-negative bacteria [41]. The current study suggests the antibacterial potential of isolated compounds **2** and **3** based on preliminary screening. However, further rigorous investigations, such as broth microdilution assays, are necessary to accurately determine their antibacterial potency, including the minimum inhibitory concentration (MIC) and minimum bactericidal concentration (MBC).

### 3.4. Antidiabetic activity

High-carbohydrate meals, especially those rich in starch, are enzymatically hydrolyzed by *α*-amylase and *α*-glucosidase into simple sugars, provoking rapid blood glucose surges that contribute to diabetic complications. Consequently, inhibiting these enzymes to decrease starch digestion presents a promising therapeutic approach to mitigate postprandial hyperglycemia [9]. Prior studies have identified several α-amylase and α-glucosidase inhibitors—including acarbose, miglitol, and voglibose—which are widely used in diabetes management [42]. Therefore, our investigation focused on these enzymes to assess the antidiabetic efficacy of isolated compounds from *A. bicolor*.

**Fig 5:**
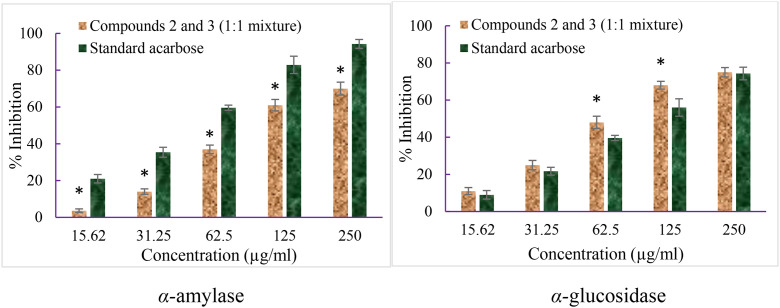
Antidiabetic activity of isolated compound and standard acarbose via α-amylase and α-glucosidase inhibitory assay. Each test was performed in triplicate (n = 3) and the data are expressed as mean ± S.D. * Significantly different from standard acarbose (at similar concentrations) at p < 0.05, using Student’s t-test.

Fig 5 illustrates the antidiabetic activity of isolated compounds. Compound **1** exhibited no enzyme inhibitory activity. In contrast, compounds **2** and **3** demonstrated dose-dependent enzyme inhibition, with EC_50_ values of 99.48 ± 3.2 µg/mL for *α-*amylase and 68.53 ± 1.7 µg/mL for *α-*glucosidase. These results are moderate compared to the standard acarbose, which showed EC_50_ values of 35.5 ± 1.2 µg/mL for *α-*amylase and 189.53 ± 1.97 µg/mL for *α-*glucosidase. Previous research by Bimal et al. reported an EC_50_ of 57.37 ± 0.9 µg/mL for quercetin against *α*-amylase using the same assay protocol, highlighting the flavonoid nucleus as the key pharmacophore responsible for enzyme inhibition [14]. However, despite these enzyme inhibitory activities, further therapeutic validation through animal experiment is necessary to confirm their antidiabetic potential.

### 3.5. Computational study

#### 3.5.1. Molecular docking.

Molecular docking is a widely computational technique in drug discovery that helps to elucidate ligand-target interactions and facilitate the design of novel therapeutics [43]. In this study, *α*-amylase and *α*-glucosidase enzymes were selected as targets for *in silico* antidiabetic investigations due to their well-established roles in diabetes pathogenesis and therapy. These enzymes catalyze the breakdown of dietary starch into absorbable monosaccharides, leading to rapid blood glucose spikes and associated diabetic complications [10,11]. Therefore, inhibiting *α*-amylase and *α*-glucosidase is a promising strategy for developing new antidiabetic agents [24]. Recent advancements have emphasized the use of *in silico* methods to identify natural product-derived inhibitors of these enzymes [26,44].

In this study, bioactive phytoconstituents isolated from *A. bicolor* were docked using the AutoDock Vina software to predict their binding sites, energies, and orientations against these enzymes. This analysis corroborates the observed *in vitro* inhibitory activity and offers exploratory insight into the molecular mechanisms underlying the inhibition of these antidiabetic enzyme [11].

**Fig 6:**
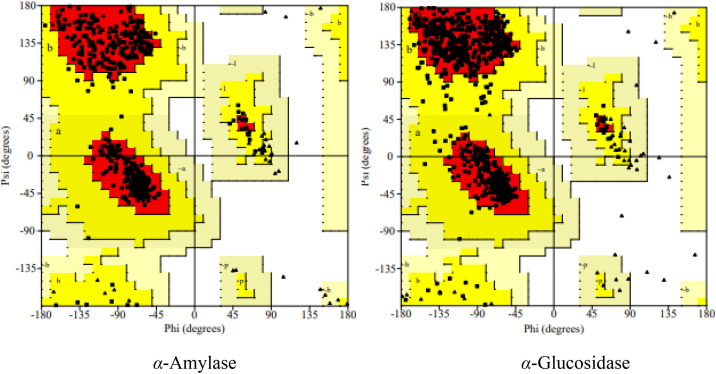
Ramachandran plot for validation of target proteins *α*-amylase and *α*-glucosidase. Amino acids are represented by black dots, with those in the red regions (A, B, L) denoting the most favored conformations. Residues located within the yellow zones (a, b, l, p) correspond to additionally allowed conformations, while those in the grey areas (~a, ~ b, ~ l, ~ p) represent generously permitted conformations. The white regions mark disallowed conformations, indicating unfavorable stereochemistry for docking analyses. Proline and glycine residues are uniquely illustrated as triangles.

Target protein validation was performed using Ramachandran plot analysis generated by the PROCHECK tool, which assesses the stereochemical quality of protein structures [29], as shown in Fig 6. For human pancreatic *α*-amylase (495 amino acids) and *α*-glucosidase (850 amino acids), over 90% of residues occupied the most favorable regions, with no residues in disallowed regions, thereby confirming the reliability of the selected protein templates [11].

**Fig 7:**
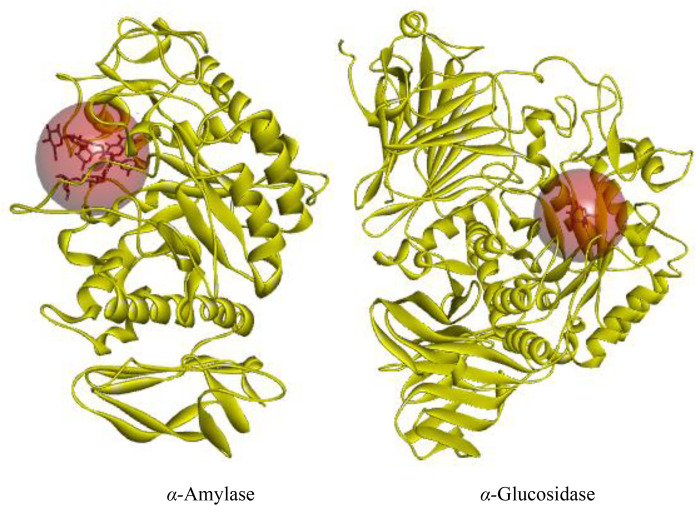
3D structure of the antidiabetic target proteins *α*-amylase and *α*-glucosidase. Co-crystal ligand bound at catalytic pocket representing the active site by a spherical grid.

The active sites of the enzymes were identified using BIOVIA Discovery Studio by analyzing the positions of co-crystallized ligands to determine the catalytic residues within their three-dimensional structures (Fig 7). For *α*-amylase, key catalytic residues included ASP 197, GLU 233, and ASP 300, while for *α*-glucosidase, important residues comprised ASP 404, ASP 518, ARG 600, ASP 616, and HIS 674. These residues are essential for enzyme activity and were used to define the docking search regions [11,14].

**Fig 8:**
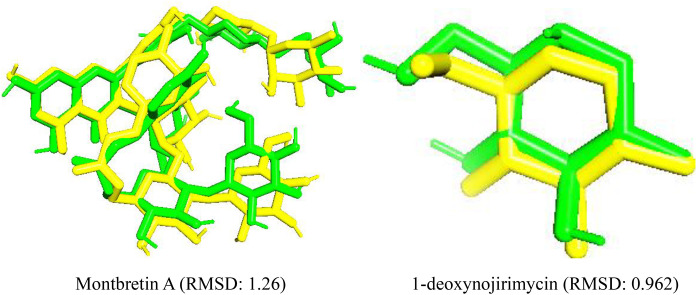
Validation of docking protocol. Superposition of docked pose (green) and native pose (yellow) of co-crystal ligands bound with ***α*** -amylase and ***α*** -glucosidase, respectively.

The validation of the docking protocol was confirmed by calculating the root-mean-square deviation (RMSD) values, with results below 2 Å indicating high accuracy. The current protocol demonstrated an RMSD of less than 2 Å when comparing native and docked co-crystal ligand confirmations (Fig 8), affirming the method’s reliability and precision [29,45].

**Fig 9:**
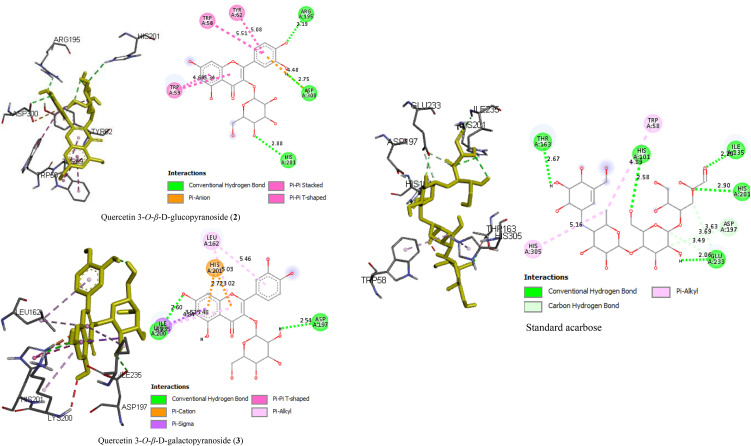
3D and 2D molecular interaction of quercetin 3-*O*-*β*-D-glucopyranoside (2), quercetin 3-*O*-*β*-D-galactopyranoside (3) and standard acarbose against *α*-amylase.

**Fig 10:**
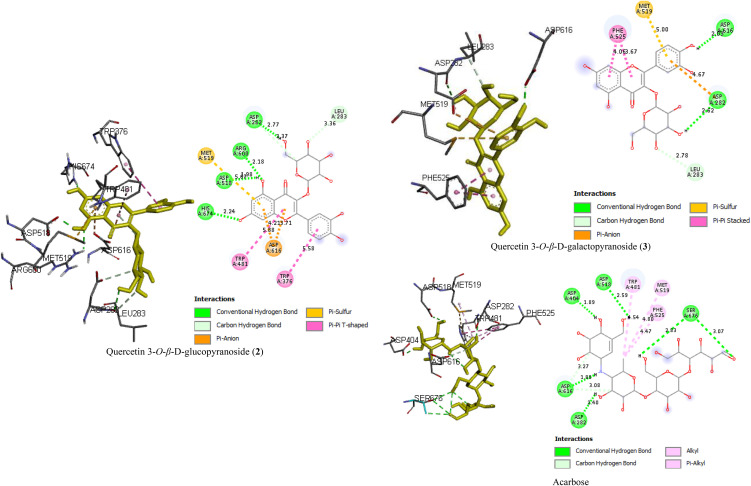
3D and 2D molecular interaction of quercetin 3-*O*-*β*-D-glucopyranoside (2), quercetin 3-*O*-*β*-D-galactopyranoside (3) and standard acarbose against *α*-glucosidase.

Docking results prioritized ligands exhibiting strong negative Gibbs free binding energies, robust hydrogen bonding, and short bond lengths [11]. Specifically, bioactive compounds **2** and **3** interacted with catalytic residues, displaying binding energies from −8.3 and −8.1 kcal/mol against *α*-amylase, and −7.2 and −6.7 kcal/mol against *α*-glucosidase. These values were comparable to those of the reference drug acarbose (−6.9 and −7.1 kcal/mol, respectively), as summarized in Table 3. Analysis of the molecular interactions revealed various types of bonds with active site residues, including conventional hydrogen bonds, carbon hydrogen bonds, pi-sigma interactions, pi-pi T-shaped interactions, pi-donor hydrogen bonds, pi-alkyl interactions, and π-π stacking, with hydrogen bonds being predominant (Fig 9 and Fig 10). These interactions suggest the formation of stable ligand-enzyme complexes [29] and support the *in vitro* enzyme inhibitory potential of the isolated compounds. However, as early-stage screening data, these findings require further pharmacological validation through comprehensive molecular, cellular, and *in vivo* studies to establish these compounds as potential antidiabetic lead candidates [8,9,46].

**Table 3:** Gibbs free binding energies (kcal/mol) and molecular interactions acquired between *A. bicolor* phytoconstituents and the antidiabetic targets *α*-amylase and *α*-glucosidase enzyme.

Ligands	*α* -amylase	*α* -glucosidase
	Docking score (active site residue interaction)	Docking score (active site residue interaction)
Compound **2**	−8.3 (ASP 300)	−7.2 (ASP 404, ASP 518, MET 519, ARG 600, ASP 616, HIS 674)
Compound **3**	−8.1 (ASP 197)	−6.7 (MET 519, ASP 616)
Acarbose	−6.9 (ASP 197, GLU 233)	−7.1 (ASP 404, ASP 518, MET 519, ASP 616, HIS 674)

#### 3.5.2. ADMET analysis.

Reliable prediction of absorption, distribution, metabolism, excretion (ADME), and toxicity profiles is essential for ensuring the safety and therapeutic effectiveness of bioactive phytoconstituents throughout the drug discovery and development processes [11,23]. The integration of *in silico* approaches offers a preliminary means to forecast these pharmacokinetic and toxicological parameters. Such methods provide notable advantages, including reduced costs, faster screening processes, environmental benefits, and a decrease in animal testing [21].

Table 4 delineates the ADME (Absorption, Distribution, Metabolism, and Excretion) attributes of bioactive compounds isolated from *A. bicolor*. Compound 1 conforms to Lipinski’s Rule of Five criteria, including a molecular weight ≤ 500 g/mol, Log P ≤ 5, hydrogen bond acceptors ≤ 10, hydrogen bond donors ≤ 5, and TPSA ≤ 140 Å^2^, indicating favorable pharmacokinetic properties related to solubility and membrane permeability [11,23]. In contrast, compounds **2** and **3** violate Lipinski’s Rule of Five, as their TPSA values, hydrogen bond acceptors, and hydrogen bond donors exceed the recommended thresholds. However, their strong *in vitro* activity suggests they serve as excellent starting points. Medicinal chemistry optimization can help improve their ADMET (Absorption, Distribution, Metabolism, Excretion, and Toxicity) profiles and overall drug-likeness [8,9,46].

**Table 4:** ADMET analysis of isolated compounds from *A. bicolor* and standard acarbose.

Parameters	Compound 1	Compound 2	Compound 3	Acarbose
***Physiochemical properties***				
Mol. Wt. (g/mol)	480.63	464.38	464.38	645.6
Number of rotatable bonds	5	4	4	13
Number of H-bond acceptors	7	12	12	19
Number of H-bond donors	6	8	8	14
Molar refractivity	129.74	110.16	110.16	137.92
TPSA (Å2)	138.45	210.51	210.51	329.01
***Lipophilicity***				
Log *P*_o/w_ (iLOGP)	2.87	1.45	1.45	0.17
Log *P*_o/w_ (XLOGP3)	0.46	0.36	0.36	−8.82
Log *P*_o/w_ (WLOGP)	1.85	−0.54	−0.54	−8.72
Log *P*_o/w_ (MLOGP)	1.13	−2.59	−2.59	−7.45
Log *P*_o/w_ (SILICOS-IT)	2.43	−0.59	−0.59	−6.36
Consensus Log *P*_o/w_	1.75	−0.38	−0.38	−6.24
***Water solubility***				
Log S (ESOL)	−2.78 (Soluble)	−3.04 (Soluble)	−3.04 (Soluble)	2.57 (Highly soluble)
Log S (Ali)	−2.94 (Soluble)	−4.35 (Moderately soluble)	−4.35 (Moderately soluble)	2.69 (Highly soluble)
Log S (SILICOS -IT)	−2.33 (Soluble)	−1.51 (Soluble)	−1.51 (Soluble)	6.23 (Soluble)
***Pharmacokinetics***				
GI absorption	High	Low	Low	Low
BBB permeability	No	No	No	No
p-gp substrate	Yes	No	No	Yes
CYP1A2 Inhibitor	No	No	No	No
CYP2C19 Inhibitor	No	No	No	No
CYP2C9 Inhibitor	No	No	No	No
CYP2D6 Inhibitor	No	No	No	No
CYP3A4 Inhibitor	No	No	No	No
Log *k*_*p*_ (Skin permeation; cm/s)	−8.91	−8.88	−8.88	−16.5
***Drug-likeness***				
Lipinski	Yes; 1 violation	No; 2 violation	No; 2 violation	No; 3 violation
Bioavailability Score	0.55	0.17	0.17	0.17
PAINS #alerts	0	1	1	0
Lead likeness #violation	No	No	No	No
Synthetic Accessibility	6.36	5.32	5.32	7.25
***In silico toxicity***				
Ames toxicity	No	No	No	No
Oral rat acute toxicity (LD_50_) (mol/kg)	2.671	2.541	2.541	2.673
Max. tolerated dose human (log mg/kg/day)	−0.214	0.569	0.569	0.613
Predicted toxicity in rodent (LD_50_: mg/kg)	9000; class 6	5000; class 5	5000; class 5	2000; class 4
Hepatotoxicity (Probability in rodent)	Inactive (0.74)	Inactive (0.82)	Inactive (0.82)	Active (0.50)
Nephrotoxic (Probability in rodent)	Inactive (0.69)	Active (0.76)	Active (0.76)	Active (0.85)
Cytotoxicity (Probability in rodent)	Inactive (0.76)	Inactive (0.69)	Inactive (0.69)	Inactive (0.69)
Carcinogenicity (Probability in rodent)	Active (0.76)	Inactive (0.85	Inactive (0.85)	Inactive (0.84)
Immunotoxicity (Probability in rodent)	Active (0.93)	Active (0.66)	Active (0.66)	Active (0.91)
Mutagenic (Probability in rodent)	Inactive (0.74)	Inactive (0.76)	Inactive (0.76)	Inactive (0.76)

Regarding *in silico* toxicity assessment, all three compounds appeared non-toxic concerning Ames mutagenicity, hepatotoxicity, and cytotoxicity. However, compounds **2** and **3** showed a moderate likelihood of nephrotoxicity, with a maximum probability of 0.76. Consequently, comprehensive molecular, cellular, genetic, and *in vivo* investigations are essential to further refine their toxicological profiles before considering these compounds as viable bioactive lead candidates.

## 4. Conclusion

This study provides initial insights into the bioactive compounds present in *A. bicolor* extract, identifying three compounds—20-hydroxyecdysone (**1**), quercetin 3-*O*-*β*-D-glucopyranoside (**2**), and quercetin 3-*O*-*β*-D-galactopyranoside (**3**)—from its 70% methanol extract for the first time. *In vitro* assays indicated that quercetin derivatives exhibited notable DPPH scavenging activity and moderate inhibitory effects on *α*-amylase and *α*-glucosidase enzymes, which support traditional uses of *A. bicolor* in managing diabetes. *In silico* molecular docking further suggested potential enzyme interactions, with favorable binding energies observed for compounds **2** and **3**. However, ADMET predictions raised concerns regarding drug-likeness and potential nephrotoxicity for these compounds, underscoring the importance of further medicinal chemistry modifications and comprehensive pharmacological studies. Additional cell-based and *in vivo* investigations are necessary to validate their therapeutic potential and assess safety profiles before considering them as promising candidates for antidiabetic phytomedicine.

### 4.1. Limitation of the study

This study primarily focused on the isolation of compounds and preliminary *in vitro* and *in silico* assessments of their biological activities, due to limitations in laboratory facilities and technological resources. These constraints may impact the robustness and generalizability of the findings related to the isolated compound’s antidiabetic potential. Furthermore, the limited quantities of isolated compounds restricted the scope of detailed evaluations, particularly in confirming their antibacterial potency through minimum inhibitory concentration (MIC) and minimum bactericidal concentration (MBC) determinations.

## Supporting information

S1 File The ^1^H, ^13^C, and DEPT-135 NMR spectra for the isolated compounds (S1–S6 Figs). (DOCX)

S2 File Graphical abstract. (DOCX)
