# Supplementary material for: Bioassay-guided isolation of antioxidant, antibacterial, and antidiabetic compounds from Aleuritopteris bicolor of Nepal: In vitro/in silico study
Source: PLoS One. 2026 Jul 30;21(7):e0354665. doi: 10.1371/journal.pone.0354665 (PMC13422865; doi:10.1371/journal.pone.0354665)
Supplement: S1 File — (DOCX) [file pone.0354665.s001.docx]

**Bioassay-guided isolation of antioxidant, antibacterial, and antidiabetic compounds from *Aleuritopteris bicolor* of Nepal: *In vitro/in silico* study**

Rekha Bhandari^1^, Sadikshya Sapkota^1^, Peru Kumari Bishwakarma^1^, Shailendra Kumar Sharma^1^, Ram Kishor Yadav^1^*****_,_ Sandesh Poudel^1^, Siddha Raj Upadhyaya^2^, Ganga Ram Upadhayay^3^, Sajan L Shyaula^3^ and Khem Raj Joshi^1^

1 School of Health and Allied Sciences, Pokhara University, Pokhara-30, Nepal

2 Central Department of Chemistry, Tribhuvan University, Katmandu, Nepal

3 Nepal Academy of Science and Technology, Lalitpur, Nepal

*****Correspondence: [hemy25869@gmail.com](mailto:hemy25869@gmail.com)

Supporting spectroscopic data


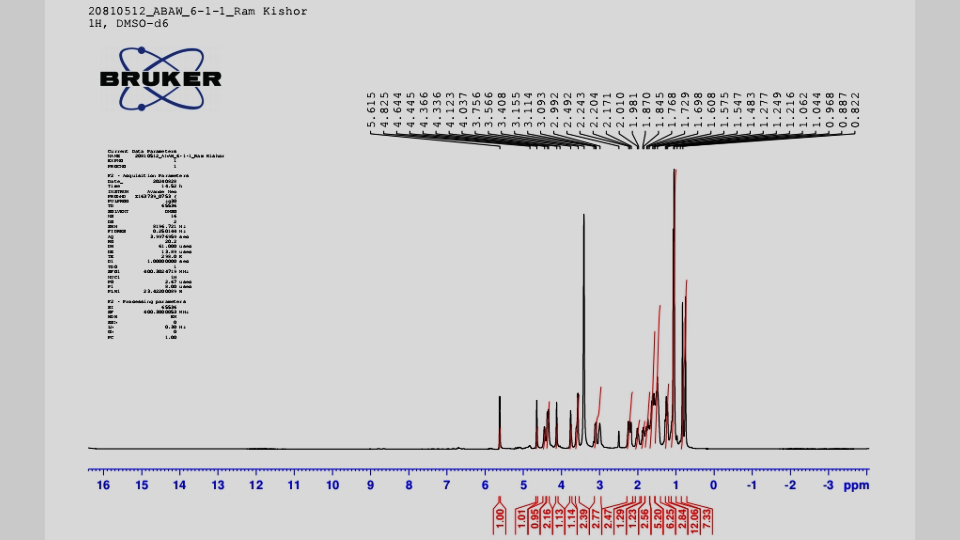


**S1 Fig.** **^1^H NMR spectra of compound 1.**

**
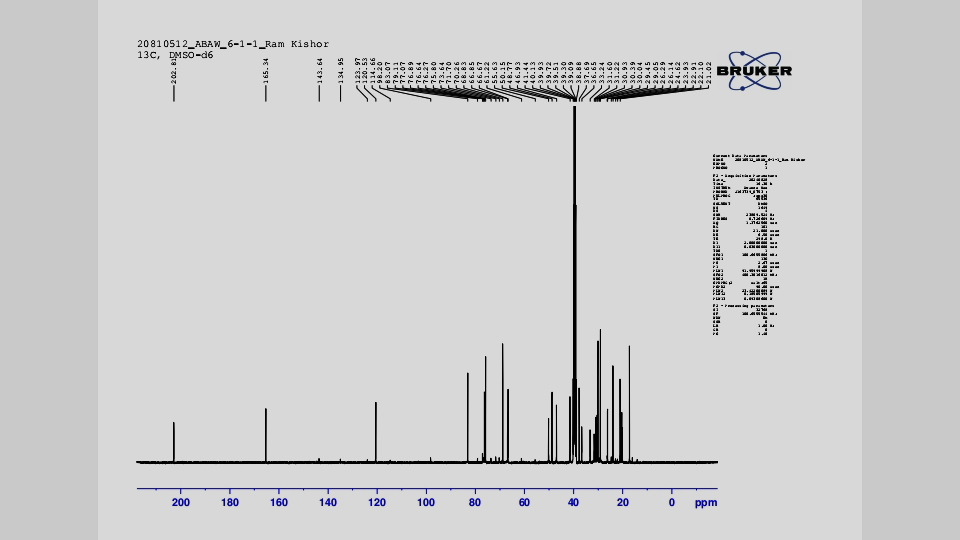
**

**S2 Fig.** **^13^C NMR spectra of compound 1.**


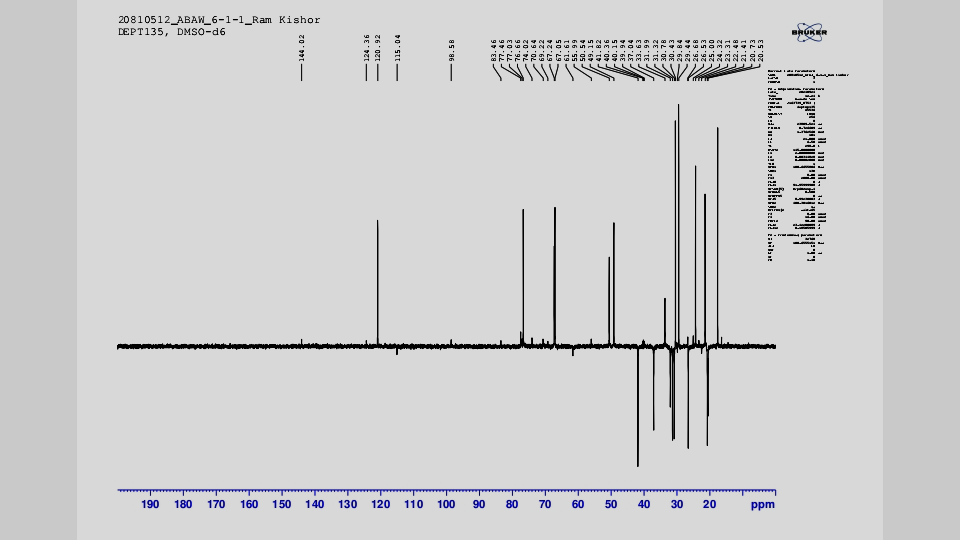


**S3 Fig.** **DEPT-135 NMR spectra of compound 1.**


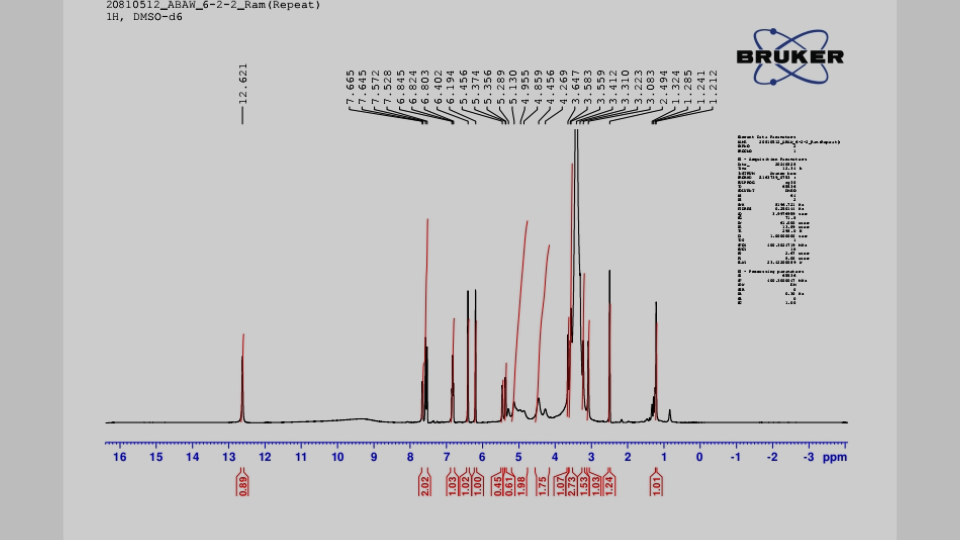


**S4 Fig.** **^1^H NMR spectra of compounds 2 and 3**.


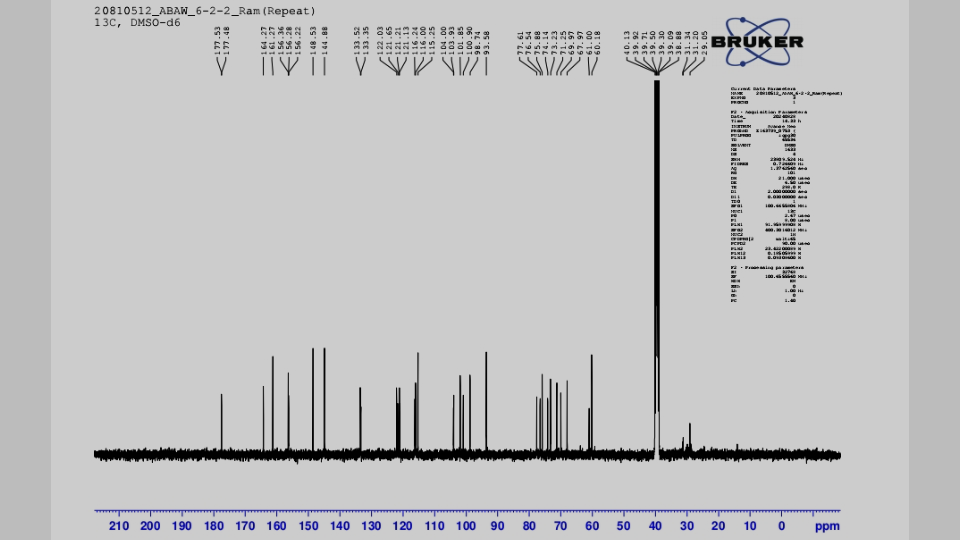


**S5 Fig. ^13^C NMR spectra of compounds 2 and 3.**

**
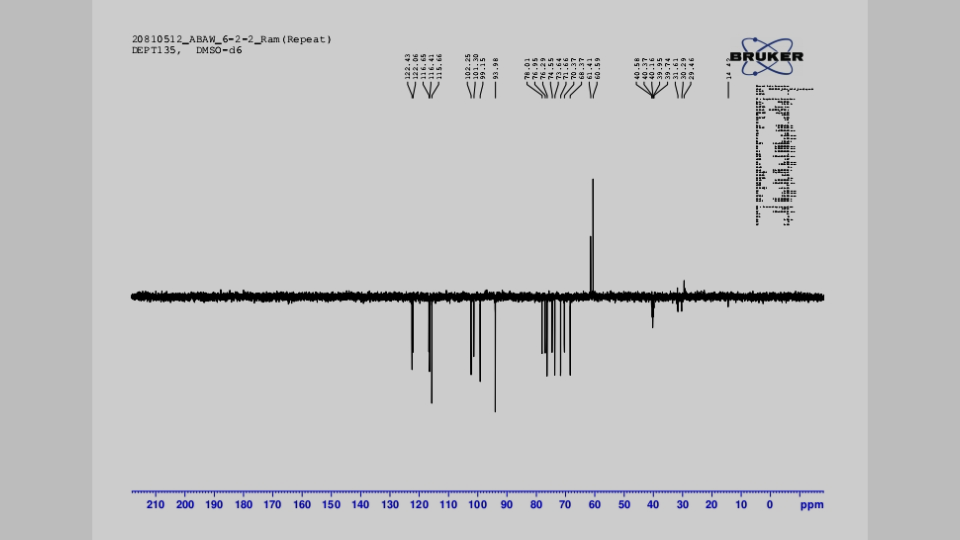
**

**S6 Fig.** **DEPT NMR spectra of compounds 2 and 3**.

.
