# Supplementary figures and images for: Bioassay-guided isolation of antioxidant, antibacterial, and antidiabetic compounds from Aleuritopteris bicolor of Nepal: In vitro/in silico study
Source: PLoS One. 2026 Jul 30;21(7):e0354665. doi: 10.1371/journal.pone.0354665 (PMC13422865; doi:10.1371/journal.pone.0354665)

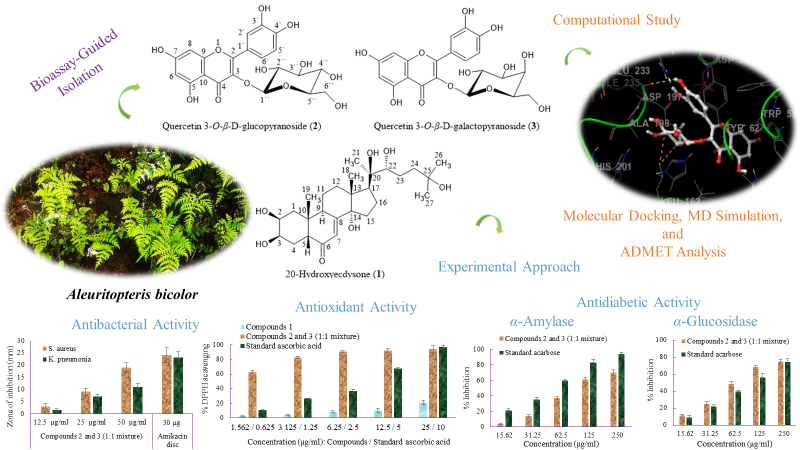

Supplement: S2 File — (DOCX) [file pone.0354665.s002.docx]
